# Microbiota composition of *Drosophila* and their environments in Utah, USA, orchards

**DOI:** 10.1128/mra.01022-25

**Published:** 2026-01-29

**Authors:** Amanda Morrison, Aubrey Cluff, Sarah Gottfredson Morgan, Emma K. Davis, Connor Hough, John M. Chaston

**Affiliations:** 1 Department of Plant and Wildlife Sciences, Brigham Young University 172811, Provo, Utah, USA; University of Wisconsin-Madison, Madison, Wisconsin, USA

**Keywords:** *Drosophila melanogaster*, Utah

## Abstract

We present a marker gene analysis of the microbiota of *Drosophila* and their fruit and soil environments, collected across a latitudinal gradient in Utah, USA. Collections varied according to fly species, sex, and starvation condition, providing a snapshot of the covariation of these variables with microbiota composition in the wild.

## ANNOUNCEMENT

To better understand the relationship between microbiota composition, latitude, and life history in *Drosophila melanogaster*, we collected and analyzed the microbiota in flies across a latitudinal gradient in Utah, USA. Some samples of starved male *D. melanogaster* are reported in a companion article (1), and the remaining 609 samples are reported here.

In Fall 2020, we performed collections at five orchards in Utah, USA (Table S10 in reference 1). At 7–13 locations throughout each orchard, we sampled 5–15 flies from individual fallen peaches via aerial net, adjacent cores of rotten and fresh peach flesh (1/4” × 1/2”), and soil (1/2” × 3/4”). Samples were immediately stored on dry ice or, for some flies, starved for ~4 h, then stored long-term at −80°C. Individual flies were examined by microscopy to retain only *D. melanogaster* and *Drosophila simulans* using established criteria (2–4), such as the presence of a distinct genital arch in male *D. simulans* and pigmentation band variation in females. Female morphological differences are not conclusively diagnostic and should be interpreted with caution.

All sample preparation steps followed manufacturer instructions unless noted otherwise. DNA was extracted using the Zymo Quick-DNA Fecal/Soil Microbe 96 Kit (D6011). Single flies, 20 mg soil, or 50 mg peach flesh was homogenized in 96-well homogenization tubes for 2 minutes at 1,750 RPM on a SPEX GenoGrinder. 16S rRNA V4 marker gene sequencing was performed using a dual-barcoding approach (5). PCR reactions were normalized and pooled (96 samples/group) using the Just-a-Plate normalization kit (Charm Biotech, JN-120-10) and concentrated (Zymo gDNA Clean & Concentrator 11-302C). Fragments (250–450 bp) were selected on a Sage Science Blue Pippin, pooled in equimolar volumes, and sequenced using Illumina MiSeq 500 Cycle v.2 chemistry. Together, we obtained 16.1 million reads that passed default Illumina quality filters in two separate runs.

**Fig 1:**
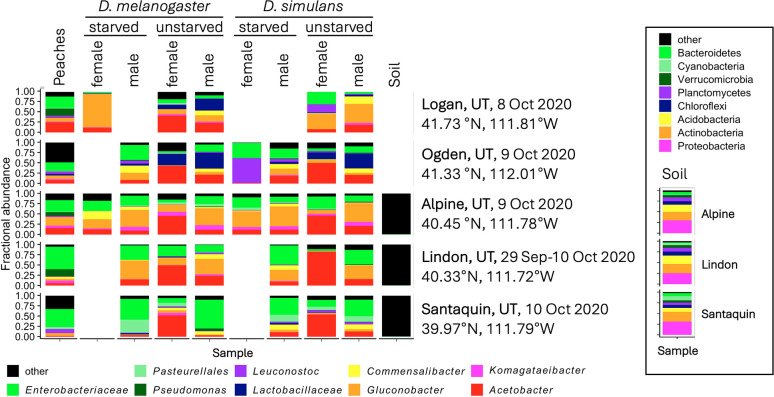
Microbiota composition of wild flies and their environments. Reads were rarefied to 146 reads per sample, clustered at the genus level, aggregated by the metadata. Genera with <1.5% overall abundance are clustered in the “other” category. The inset shows phylum-level taxonomic assignments to the soil samples, first rarefied to 8,300 reads per sample.

Sequences were denoised, dereplicated, and assigned as amplicon sequence variants, and OTU tables were created as described previously using QIIME2 2022.11 with DADA2, all using default parameters (1, 6–13). Taxonomic assignments were made using GreenGenes 13_8_99 (8). Removing samples reported previously or which are irrelevant to this work yielded, before/after filtering out *Wolbachia* reads, respectively, 12,860,107/2,833,611 reads in 609/600 samples (minimum 6/2; maximum 194,940/194,715; median 13,129/745; mean 21,117/4,723) (Fig. 1). Bray–Curtis distances varied with sampling location, substrate, fly species, sex, and starvation condition (Table 1). ANCOM identified bacterial genera that varied between conditions (14) (Table 1). These fruit, soil, and fly samples provide additional insight into diet- and geography-dependent variations in microbiota composition of wild *Drosophila*.

**TABLE 1:** PERMANOVA and ANCOM analysis

	Df	SS	*R* ^2^	*F*	*P*	Genera that differ by ANCOM
All samples						
Substrate	2	7.31	0.04	10.51	<10^−3^	*Acetobacter*, *Gluconobacter*, *Pseudomonas*, unassigned Enterobacteriaceae
Location	4	19.05	0.1	13.69	<10^−3^	*Gluconobacter*, *Komagataeibacter*, unassigned Enterobacteriaceae, unassigned Lactobacillaceae, unassigned Pasteurellales,
Residual	473	164.57	0.86			
Total	479	190.94	1			
Fly samples						
Species	1	0.84	0.01	2.67	<10^−3^	None
Starvation condition	1	6.04	0.04	19.19	<10^−3^	*Acetobacter*, *Gluconobacter*, unassigned Lactobacillaceae
Sex	1	5.75	0.04	18.25	<10^−3^	*Acetobacter*
Location	4	17.12	0.11	13.59	<10^−3^	*Gluconobacter*, *Komagataeibacter*, *Leuconostoc*, unassigned Enterobacteriaceae, unassigned Lactobacillaceae, unassigned Pasteurellales
Residual	407	128.17	0.81			
Total	414	157.93	1			

## Data Availability

The sequences used in this study were deposited in the Sequence Read Archive under BioProject accession number PRJNA1306182 and BioSample accession numbers SAMN51356473-SAMN51357108.
